# Microwave-assisted Synthesis of an Important Intermediate of Benazepril

**DOI:** 10.4103/0250-474X.70489

**Published:** 2010

**Authors:** B Mistry, Dipti Medhane, Krishnapriya Mohanraj, Sanjeevani A. Ghone

**Affiliations:** Department of Pharmaceutical Chemistry, Bombay College of Pharmacy, Kalina, Santacruz (E) Mumbai - 400 098, India; 1 School of Pharmacy & Technology Management, SVKM’s NMIMS, Vile Parle (W), Mumbai - 400 056, India

**Keywords:** Benazepril intermediate, microwave assisted synthesis

## Abstract

Rapid and efficient methods for the synthesis of an important intermediate of benazepril ethyl 3-phthalimido-2,3,4,5-tetrahydro-1*H*-[1]benzazepin-2-one-1-acetate under the influence of microwave irradiation are described. A comparative study of conventional and microwave assisted method is briefly discussed.

Benazepril is an orally active non-sulfhydryl containing long acting ACE inhibitor which inhibits the enzymatic conversion of angiotensin-I to the vasoconstrictor, angiotensin-II. Retrosynthesis of benazepril (fig. 1) revealed ethyl 3-amino-2,3,4,5-tetrahydro-1*H*-[1]-benzazepin-2-one-1-acetate to be an important synthon which can be easily prepared from ethyl 3-phthalimido-2,3,4,5-benzazepin-2-one-1-acetate[1].

**Fig. 1:**
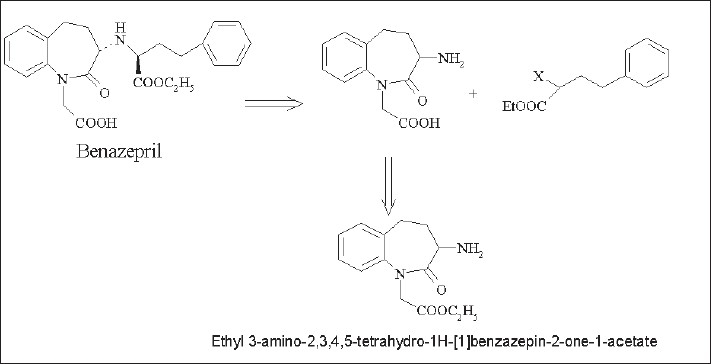
Retrosynthesis of benazepril

Microwave-assisted synthesis has many advantages as they can accelerate reaction rates, and yields can be improved[2]. In various literature reports, synthesis of this intermediate of benazepril using conventional thermal methods involved long reaction times[3–5]. The ability of microwave irradiation to reduce reaction time led to an idea for this synthesis to be carried out under the influence of microwave irradiation.

The present work involves study of the synthesis of ethyl 3-phthalimido-2,3,4,5-tetrahydro-1*H*-[1]-benzazepin-2-one-1-acetate (4) from 3-bromo-2,3,4,5-tetrahydro-1*H*-1-benzazepin-2-one (1) via two routes A and B using conventional thermal conditions and microwave (MW) irradiation (fig. 2). In route A, (1) was treated with potassium phthalimide using DMF as the solvent to prepare 3-phthalimido-2,3,4,5-tetrahydro-1*H*[1]-benzazepin-2-one (2). It was then treated with ethyl bromoacetate in presence of sodium *t*-butoxide in DMF to prepare (4). In route B, (1) was treated with ethyl bromoacetate in presence of sodium *t*-butoxide to prepare 3-bromo-1-ethoxycarbonylmethyl-2,3,4,5-tetrahydro-1*H*-1-benzazepin-2-one (3) which was further treated with potassium phthalimide in DMF to prepare (4). All these reactions were carried out by conventional and microwave-assisted methods. Microwave-assisted reactions were carried out in a domestic microwave oven at 70W. The microwave-assisted synthesis resulted in drastic reduction in reaction time (Table 1). The yields of all the reactions were relatively similar. Formation of compound (3) from (1) occurred only under microwave irradiation while conventionally no product was formed even under more drastic conditions. Hence, conversion of compound (4) from (3) was not carried out under conventional thermal conditions. All the reactions were monitored by thin layer chromatography for completion and for establishing purity. All the synthesized compounds were characterized by melting points and spectral properties (FT-IR and ^1^HNMR spectra).

**TABLE 1:** COMPARISON OF SYNTHESIS OF COMPOUNDS 2, 3 AND 4 BY CONVENTIONAL (THERMAL) AND MICROWAVE (MW) IRRADIATION METHODS

Reactant	Product	Yield %		Time		Melting point of products
		Thermal	MW	Thermal (h)	MW (m)	
(1)	(2)	84 %	77 %	29 h	3 m	225°
(2)	(4)	65 %	67 %	18 h	4 m	96°
(1)	(3)	No product formed	85 %	24 h	4 m	114°
(3)	(4)	-	65 %	-	4m	96°

(1) is 3-Bromo-2,3,4,5-tetrahydro-1*H*-[1]-benzazepin-2-one, (2) is 3-phthalimido-2,3,4,5-tetrahydro-1*H*-[1]-benzazepin-2-one, (3) is 3-bromo- 1-ethoxycarbonylmethyl-2,3,4,5-tetrahydro-1*H*-[1]-benzazepin-2-one and (4) is ethyl 3-phthalimido-2,3,4,5-tetrahydro-1*H*-[1]-benzazepin-2-one-1-acetate

**Fig. 2:**
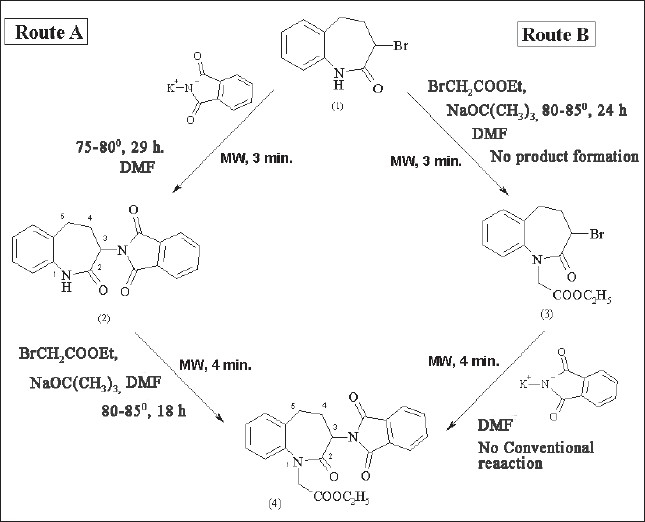
Scheme for synthesis of ethyl 3-phthalimido-2,3,4,5-tetrahydro-1*H*-1-benzazepin-2-one-1-acetate (4) (1) is 3-Bromo-2,3,4,5-tetrahydro-1*H*-[1]-benzazepin-2-one, (2) is 3-phthalimido-2,3,4,5-tetrahydro-1H-[1]-benzazepin-2-one, (3) is 3-bromo-1-ethoxycarbonylmethyl-2,3,4,5-tetrahydro-1*H*-[1]-benzazepin-2-one and (4) is ethyl 3-phthalimido-2,3,4,5-tetrahydro-1*H*-[1]-benzazepin-2-one-1-acetate

Conventional synthesis of compounds (2) and (4) were carried out as follows. To a suspension of 10.3 g (0.046 mol) of compound (1) in 25 ml of anhydrous DMF, 9.9 g (0.0527 mol) of potassium phthalimide in 15 ml of anhydrous DMF was added. The suspension was stirred for 29 h at 75-80°, cooled to room temperature and DMF was removed under vacuum. It was then extracted with 3×25 ml ethyl acetate. The combined ethyl acetate extract was washed with water and dried over anhydrous magnesium sulphate. Evaporation of solvent under vacuum yielded compound (2). Yield: 12 g (84%) Melting point: 225° FT-IR: 3065 cm^-1^(C-H stretch, aromatic), 1774 cm^-1^(C=O stretch, cyclic imide), 1751 cm^-1^(C=O stretch, cyclic amide).^1^H NMR (CDCl_3_): δ 8.15 (broad singlet, 1H, -NH), 7.74-7.91 (m, 8H, Aromatic protons), 2.80 (t, 1H, -CH (C-3)), 2.38 (t, 2H, -CH_2_(C-5)), 2.20-2.32 (m, 2H, -CH_2_(C-4)).

To a suspension of 2 g (0.0098 mol) of compound (2) in 25 ml of anhydrous DMF, a solution of 1.05 g (0.011 mol) of sodium *t*-butoxide in 5 ml of anhydrous DMF was added. To this, 1.82 g (0.011 mol) of ethyl bromoacetate was added and resulting suspension was stirred for 1 h followed by heating at 80-85° for 18 h. It was cooled to room temperature and poured into 25 ml of cold water. The suspension was filtered and the residue was washed with 35 ml DMF-water (4:1) and 10 ml cold water and dried to yield compound (4). Yield: 1.66 g (65 %) Melting point: 96° FT-IR: 1772 cm^-1^(C=O stretch, cyclic imide), 1743cm^-1^(C=O stretch, cyclic imide), 1718 cm^-1^(C=O stretch, ester). ^1^HNMR (CDCl_3_): δ 7.73-7.92 (m, 8H, Aromatic protons), 4.45 (s, 2H, -CH_2_-N), 4.23 (q, 2H, -CH_2_-CH_3_), 2.80 (t, 1H, -CH, C-3), 2.40 (t, 2H, -CH_2_, (C-5)), 2.20-2.34 (m, 2H, -CH_2_, (C-4)), 1.29(t, 3H, CH_3_-CH_2_).

Microwave assisted synthesis of compounds 2, 3 and 4 were carried out as follows. In a small pear shaped flask, a suspension of 100 mg (0.4 mmol) of compound (1) in 1 ml anhydrous DMF was treated with 95 mg (0.5 mmol) of potassium phthalimide and this reaction mixture was heated in a domestic microwave oven at 70W for 3 min. The compound (2) was obtained after similar workup as in conventional synthesis. Yield: 100 mg (77 %). IR and ^1^HNMR spectral data was found similar to as obtained in conventional synthesis

To 50 mg (1.3 mmol) of compound (2), a solution of 17.5 mg (1.4 mmol) of sodium *t*-butoxide in 2 ml anhydrous DMF was added. To this, 28.75 mg (1.4 mmol) of ethyl bromoacetate was added and resulting suspension was irradiated in conventional microwave at 70W for 4 min. The compound (4) was obtained after similar workup as given in conventional synthesis. Yield: 40 mg (67%) IR and ^1^HNMR spectral data was found similar to as obtained in conventional synthesis

To 100 mg of compound (1), a solution of 60 mg (0.6 mmol) of sodium t-butoxide in 2 ml DMF was added. To this, 0.1 ml (0.6 mmol) of ethyl bromoacetate was added. The resulting suspension was irradiated in domestic microwave oven at 70W for 4 min. Usual workup gave compound (3). Yield: 120 mg (85 %) Melting point: 114° FT-IR: 1745 cm^-1^(C=O stretch, ester), 1684 cm^-1^(C=O stretch, cyclic amide), 1705 cm^-1^(C=O stretch, ester), 555 cm^-1^(C-Br stretch).

To a suspension of 100 mg (0.32 mmol) of compound (3) in 1 ml anhydrous DMF, 70 mg of potassium phthalimide was added. The reaction mixture was irradiated in conventional microwave oven at 70W for 4 min. The compound (4) was obtained after usual workup. Yield: 80 mg (65 %). IR and ^1^HNMR spectral data was found similar to as obtained in conventional synthesis.

Microwave assisted synthesis thus provided a rapid and efficient method for synthesis of an intermediate of benazepril. Rate acceleration was observed in microwave assisted methods of synthesis.
